# One‐Step Fabrication of 0D Cs_4_PbBr_6_ Perovskite with Nonlinear Optical Properties for Ultrafast Pulse Generation

**DOI:** 10.1002/advs.202404465

**Published:** 2024-07-12

**Authors:** Ning Jiang, Hongwei Chu, Zhongben Pan, Han Pan, Shengzhi Zhao, Dechun Li

**Affiliations:** ^1^ School of Information Science and Engineering, and Key Laboratory of Laser and Infrared System of Ministry of Education Shandong University Qingdao 266237 China

**Keywords:** Cs4PbBr6/CsPbBr3 composites, Cs4PbBr6 perovskite, mode‐locking operation, nonlinear optical properties, saturable absorber

## Abstract

Low‐dimensional lead halide perovskites demonstrate remarkable nonlinear optical characteristics attributed to their distinctive physical structures and electronic properties. Nevertheless, the investigation into their nonlinear optical properties remains in its incipient stages. This study addresses this gap by precisely controlling solvent volumes to synthesize both 0D Cs_4_PbBr_6_ and Cs_4_PbBr_6_/CsPbBr_3_ perovskites. Remarkably, as saturable absorbers, both pure Cs_4_PbBr_6_ and Cs_4_PbBr_6_/CsPbBr_3_ composites exhibit favorable nonlinear optical properties within the C‐band, showcasing modulation depths of 9.22% and 16.83%, respectively. Moreover, for the first time, Cs_4_PbBr_6_ and Cs_4_PbBr_6_/CsPbBr_3_ composites have been successfully integrated into erbium‐doped fiber lasers to realize the mode‐locking operations. The utilization of the Cs_4_PbBr_6_/CsPbBr_3_ composites as a saturable absorber that enables the generation of conventional soliton mode‐locked laser pulses with a pulse duration of 688 fs, and a repetition frequency of 10.947 MHz at a central wavelength of 1557 nm. Cs_4_PbBr_6_ is instrumental in generating laser pulses at a frequency of 10.899 MHz, producing pulse widths of 642 fs at the central wavelength of 1531.2 nm and 1.02 ps at the central wavelength of 1565.3 nm, respectively. The findings of this investigation underscore the potential utility of 0D Cs_4_PbBr_6_ and Cs_4_PbBr_6_/CsPbBr_3_ composites as promising materials for optical modulation within fiber laser applications.

## Graphical abstract

This study focuses on exploring the nonlinear optical properties of 0D Cs_4_PbBr_6_ and Cs_4_PbBr_6_/CsPbBr_3_ composites perovskites. For the first time, they are successfully integrated as saturable absorbers into erbium‐doped fiber lasers, achieving conventional soliton mode‐locking operations within the C‐band.

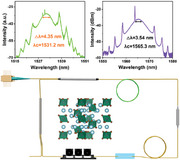

## 1 Introduction

Low‐dimensional lead halide perovskites, exemplified by CsPbX_3_ (X = Cl, Br, I), have garnered significant interest in the realm of optoelectronic devices because of their remarkable luminescent and photoelectric properties.^[^ ^1^, ^2^, ^3^, ^4^, ^5^, ^6^, ^7^, ^8^ ^]^ These perovskites are distinguished by a characteristic crystal structure wherein the [PbX_6_]^4−^ octahedron serves as the fundamental unit. Variations in the connectivity of these [PbX_6_]^4−^ octahedra give rise to diverse crystal structures, spanning from 3D to 0D.^[^ ^9^, ^10^ ^]^ The lattice structure of the traditional 3D perovskite material CsPbX_3_ is shown in Figure S1a (Supporting Information), in which the [PbX_6_]^4‐^ octahedron shares each X^−^ anion at the angle between the two octahedra, thus forming a cubic Pb─X─Pb frame. Cs atoms are located in the spaces between the octahedra, at the center of the cubic faces. As a derivative phase of CsPbX_3_, the crystal structure of the 0D Cs_4_PbX_6_ phase is different from that of the CsPbX_3_ phase. The lattice structure diagram of 0D Cs_4_PbX_6_ as shown in Figure S1b (Supporting Information) belongs to the R‐3c (167) space group. Although the [PbX_6_]^4‐^ octahedra in Cs_4_PbX_6_ are still surrounded by eight Cs atoms, the [PbX_6_]^4‐^ octahedra are separated by the surrounding Cs atoms. There are no direct connections between the octahedra, and the X^−^ anions in Cs_4_PbBr_6_ are no longer shared between the [PbX_6_]^4‐^ octahedra. As a member of the perovskite family, 0D Cs_4_PbBr_6_ has received much attention due to its unique crystal structure and photovoltaic properties.^[^ ^11^, ^12^, ^13^, ^14^ ^]^ Since the absorption edge of Cs_4_PbBr_6_ was first reported in 1983,^[^ ^15^ ^]^ researchers have extensively explored its diverse and unique properties. Theoretical calculations and experimental studies have demonstrated that 0D Cs_4_PbBr_6_ exhibits higher stability compared to 3D CsPbBr_3_.^[^ ^16^, ^17^, ^18^, ^19^ ^]^ Specifically, its binding energy of 222 meV surpasses that of 3D CsPbBr_3_ (40 meV), rendering it particularly stable in humid environments. Moreover, Cs_4_PbBr_6_ showcases a notably high photoluminescence quantum yield (PLQY); for instance, pure solid Cs_4_PbBr_6_ boasts a PLQY of 45%, markedly superior to that of 3D CsPbBr_3_. Additionally, colloidal semiconductor nanocrystals based on Cs_4_PbBr_6_ synthesized via low‐temperature reverse microemulsion exhibit excellent photoluminescence quantum yield (PLQY) of up to 65%.^[^ ^20^, ^21^ ^]^ However, the inevitable formation of the CsPbBr_3_ phase during the synthesis of Cs_4_PbBr_6_ hinders further investigation into Cs_4_PbBr_6_.^[^ ^22^ ^]^

Furthermore, low‐dimensional lead halide perovskites exhibit outstanding nonlinear optical characteristics alongside their unique physical and distinctive photoelectric characteristics.^[^ ^23^, ^24^, ^25^, ^26^, ^27^, ^28^, ^29^ ^]^ Research findings have demonstrated that 3D lead halide perovskite nanocrystals (NCs) exhibit giant two‐photon absorption (2PA) cross‐sections ranging from 10^5^ to 10^7^ GM, surpassing traditional semiconductor NCs by up to two orders of magnitude.^[^ ^30^, ^31^ ^]^ Lead halide perovskite NCs demonstrate significant single photon absorption saturation.^[^ ^32^, ^33^, ^34^ ^]^ It is noteworthy that, due to the intense enhancement of exciton resonance, 0D lead halide NCs also exhibit outstanding third‐order nonlinear optical properties and high‐order nonlinear optical responses.^[^ ^35^ ^]^ The remarkable characteristics of low‐dimensional lead halide perovskite materials position them as promising options for saturable absorbers (SA), facilitating their further application in ultrafast pulsed laser systems.

Herein, Cs_4_PbBr_6_/CsPbBr_3_ composites and pure Cs_4_PbBr_6_ perovskites were prepared by a one‐step method with the volume of solvent controlled. Their nonlinear optical properties as SAs in the C‐band were studied. The modulation depth of Cs_4_PbBr_6_ and Cs_4_PbBr_6_/CsPbBr_3_ is 9.22% and 16.83%, respectively. When Cs_4_PbBr_6_ and Cs_4_PbBr_6_/CsPbBr_3_ composites SAs are connected to Erbium‐doped fiber lasers (EDFL), stable mode‐locking and conventional soliton spectra are observed. Our results confirmed that 0D Cs_4_PbBr_6_ and Cs_4_PbBr_6_/CsPbBr_3_ could be used as new suitable SAs for ultrafast and ultranarrow fiber lasers.

## 2 Results and Discussion

### 2.1 Characterization of Cs_4_PbBr_6_/CsPbBr_3_ and Cs_4_PbBr_6_ Perovskite

**Figure 1:**
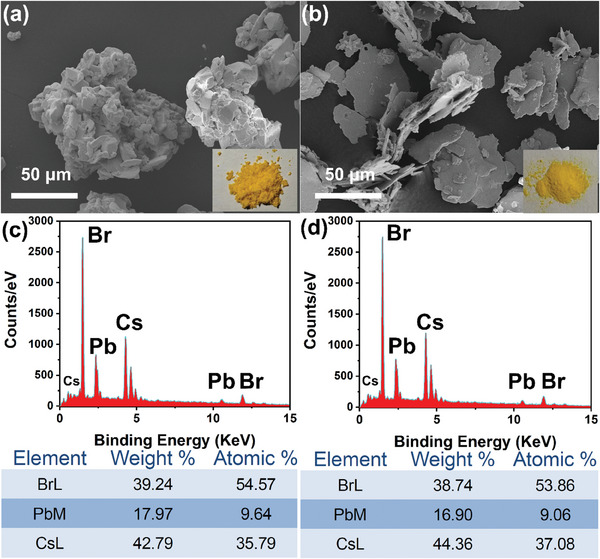
a,b) SEM images of Cs_4_PbBr_6_/CsPbBr_3_ and Cs_4_PbBr_6_ powders; c,d) The EDS point analysis spectrum and the table of relative element content Cs_4_PbBr_6_/CsPbBr_3_ and Cs_4_PbBr_6_ powders.

The present study employed a one‐step method to synthesize Cs_4_PbBr_6_/CsPbBr_3_ composites and pure Cs_4_PbBr_6_ powders, by precisely controlling the solvent volume. SEM images of Cs_4_PbBr_6_/CsPbBr_3_ composites and Cs_4_PbBr_6_ powders are shown in **Figure** **1**a,b. The Cs_4_PbBr_6_/CsPbBr_3_ composites exhibit a heterogeneous lumpy structure, characterized by varying dimensions, while the pure Cs_4_PbBr_6_ manifests a micro‐sheet architecture, with lengths extending to tens of micrometers and thicknesses ranging between 1 and 2 micrometers. The insets in Figure 1a,b demonstrate the distinctive orange color of Cs_4_PbBr_6_/CsPbBr_3_ powder and the yellow color of Cs_4_PbBr_6_ powder. The dispersion of these powders was facilitated by toluene, followed by ultrasonic treatment. Figure S2a,b (Supporting Information) depict the morphology of Cs_4_PbBr_6_/CsPbBr_3_ composites and Cs_4_PbBr_6_ after dispersion. The element ratio of Cs_4_PbBr_6_/CsPbBr_3_ and Cs_4_PbBr_6_ was assessed utilizing energy‐dispersive X‐ray spectroscopy (EDS). As shown in Figure 1c, the atomic composition indicates Cs, Pb, and Br contents of 35.79%, 9.64%, 54.57%, suggesting the presence of trace amounts of CsPbBr_3_ within the Cs_4_PbBr_6_ compound. Compared with Figure 1c, the atomic content of Cs in Figure 1d increases while the atomic content of Pb, and Br increases. The resultant atomic content ratio of 4:1:6, aligns consistently with the expected elemental composition of Cs_4_PbBr_6_, thus affirming the successful synthesis of pure Cs_4_PbBr_6_. To further pinpoint the content of CsPbBr_3_ in Cs_4_PbBr_6_/CsPbBr_3_ composites, the inductively coupled plasma mass spectrometer (ICP‐MS) was used. The contents of Cs, Pb, and Br elements of Cs4PbBr6 and Cs_4_PbBr_6_/CsPbBr_3_ composites are shown in Table S1 (Supporting Information). The amount of CsPbBr_3_ in the Cs_4_PbBr_6_/CsPbBr_3_ composites is estimated at ≈21% according to the measured Cs/Pb atomic ratio of 3.38.

**Figure 2:**
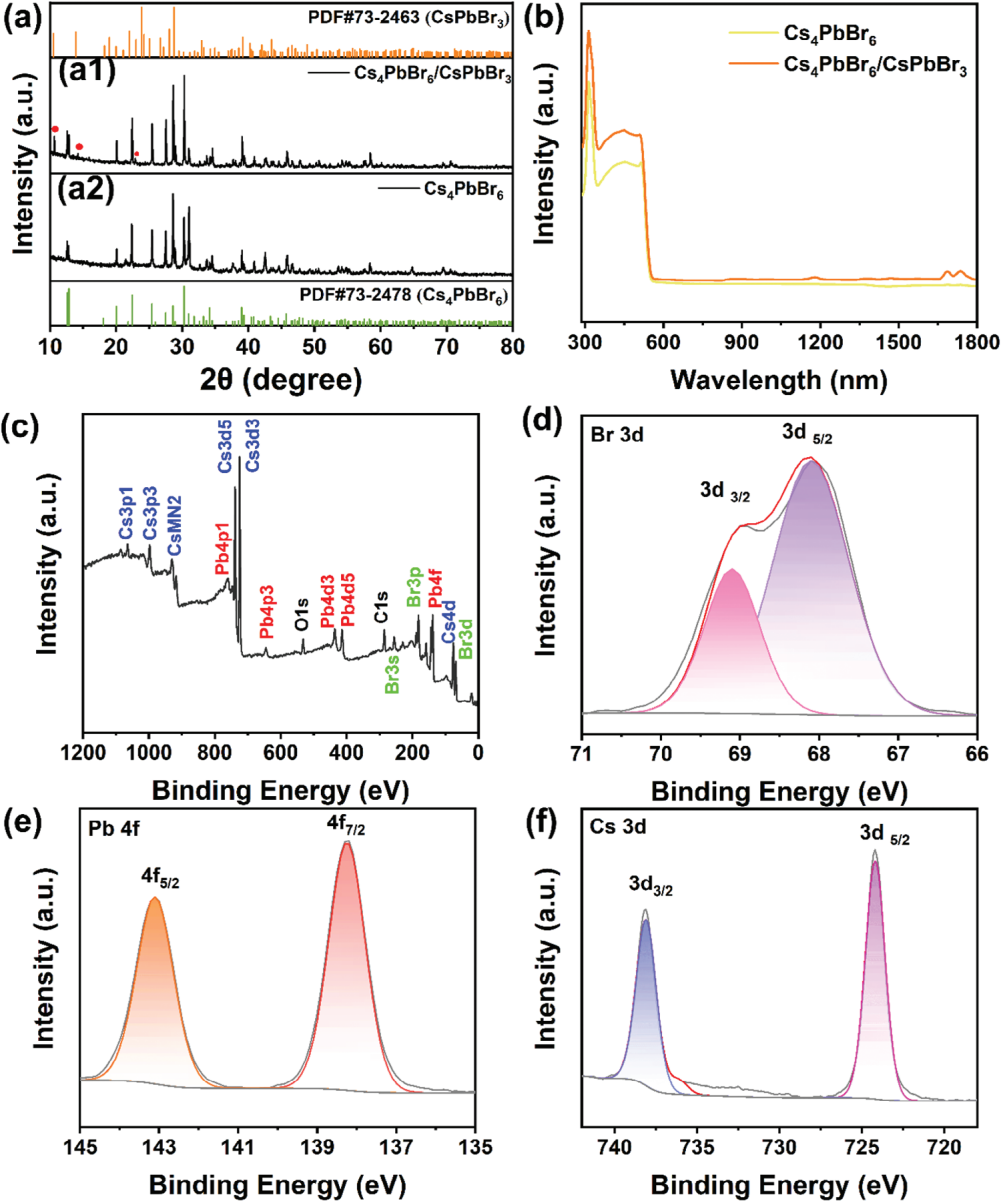
a) XRD patterns of as‐synthesized (a1) Cs_4_PbBr_6_/CsPbBr_3_ and (a2) Cs_4_PbBr_6_ powders; b) UV–vis–IR absorption spectra of Cs_4_PbBr_6_/CsPbBr_3_ and Cs_4_PbBr_6_; c–f) XPS spectrum of Cs_4_PbBr_6_ powders.

The synthesized Cs_4_PbBr_6_/CsPbBr_3_ composites and Cs_4_PbBr_6_ powder underwent characterization via X‐ray diffraction (XRD) analysis. As illustrated in **Figure** **2**a1, three additional diffraction peaks at 10.5°, 13.9°, and 23.0° were identified, highlighted in red, which corresponded to the crystal planes of CsPbBr_3_ (001), (012), and (014) respectively according to PDF#73‐2463. The remaining diffraction peaks exhibited a close concordance with the reference pattern for Cs_4_PbBr_6_ (PDF#73‐2478). By increasing the volume of solvent, the diffraction peak belonging to CsPbBr_3_ disappears (Figure 2a2), and all diffraction peaks belong to Cs_4_PbBr_6_, indicative of CsPbBr_3_ dissolution by excess DMSO while Cs_4_PbBr_6_ remains unaffected.^[^ ^20^ ^]^ This observation, in comparison with Figure 2a1, further confirms the successful synthesis of pure Cs_4_PbBr_6_ powders. The absorption spectra of Cs_4_PbBr_6_ and the Cs_4_PbBr_6_/CsPbBr_3_ powder are depicted in Figure 2b. In the wavelength range of 300–1800 nm, both materials exhibit a prominent and narrow peak at 314 nm, corresponding to the ^1^S_0_‐^3^P_1_ transition in Pb^2+^ centers within the (PbBr_6_)^4−^ octahedra of Cs_4_PbBr_6_.^[^ ^21^ ^]^ The absorption peak observed at 515 nm can be attributed to excitonic absorption in either the Cs_4_PbBr_6_ or CsPbBr_3_ phase.^[^ ^36^, ^37^ ^]^ The chemical elements and valence states of Cs_4_PbBr_6_ and Cs_4_PbBr_6_/CsPbBr_3_ were investigated using X‐ray photoelectron spectroscopy (XPS). High‐resolution XPS spectral analysis was performed on Cs 3d, Pb 4f, and Br 3d, as shown in Figure 2d–f, respectively. The Br 3d peaks at 67.8 and 68.9 eV corresponded to the electronic energy levels of Br 3d_5/2_ and Br 3d_3/2_, respectively. Figure 2e illustrates that the peaks of Cs 3d were located at binding energies of 724.2 and 738.1 eV, which can be attributed to the electronic energy levels of Cs 3d_5/2_ and Cs 3d_3/2_, respectively. The binding energy for the Pb state was determined to be at an energy level of Pb 4f_7/2_ with a value of 138.3 eV, while the binding energy for Pb 4f_5/2_ was found to be 143.2 eV.^[^ ^38^, ^39^, ^40^ ^]^ The XPS spectrum of Cs_4_PbBr_6_/CsPbBr_3_ is shown in Figure S3 (Supporting Information). Through comparison with the XPS spectrum results of Cs_4_PbBr_6_, it is found that the binding energy of Pb, Cs, and Br does not change after the addition of CsPbBr_3_. This is because the two materials Cs_4_PbBr_6_ and CsPbBr_3_ have the same element type and valence.

### 2.2 In‐line Nonlinear Optical Absorption Measurements

**Figure 3:**
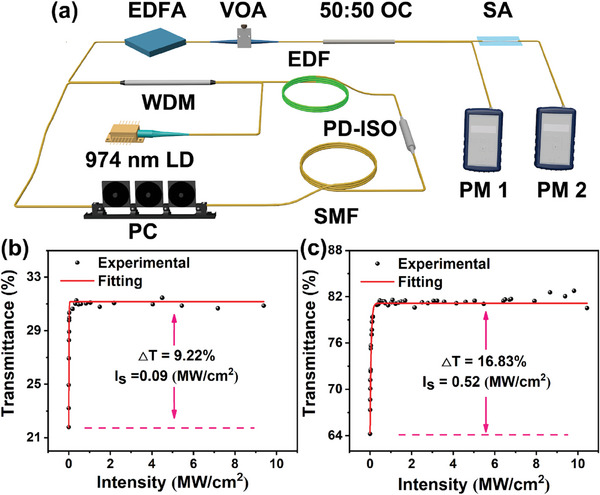
a) The schematic diagram of the twin‐detector measurement system; b,c) Nonlinear transmission curve of Cs_4_PbBr_6_ and Cs_4_PbBr_6_/CsPbBr_3_ SA.

**Figure 4:**
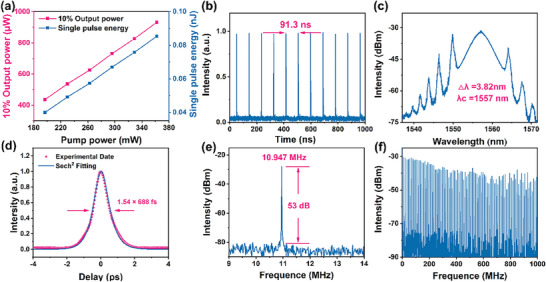
Conventional soliton mode‐locking of Cs_4_PbBr_6_/CsPbBr_3_ near 1560 nm. a) Variation of output power and single pulse energy; b) Pulse train; c) Typical optical spectrum; d) Autocorrelation trace; e) RF spectrum; f) RF spectrum within a span of 0–1 GHz.

A balanced twin‐detector measurement system was utilized to investigate the nonlinear optical properties of the Cs_4_PbBr_6_ and Cs_4_PbBr_6_/CsPbBr_3_ SA (**Figure** **3**a). The pump source utilized was a nonlinear polarization rotation (NPR) fiber laser operating at a central wavelength of 1532 nm, a pulse width of 822 fs, and a repetition frequency of 12.17 MHz. Following amplification by an erbium‐doped fiber amplifier (EDFA), the pulsed light's intensity was adjusted utilizing a variable optical attenuator (VOA). Subsequently, the light was split by a 50:50 optical coupler (OC) and directed to the power meter (PM). The nonlinear transmission characteristics, depicted as a function of excitation intensity, are illustrated in Figure 4b,c and were subjected to fitting utilizing the following Equation (1):

(1) $$T\;\left(I\right)=1-\Delta T\cdot \exp\left(\frac{-I}{I_{sat}}\right)-T_{ns}$$

where *T(I)* represents the transmission, *ΔT* denotes the modulation depth, *I* signifies the input intensity, *I_sat_* is the saturation intensity, and *T_ns_* stands for the nonsaturable loss. The modulation depth of Cs_4_PbBr_6_ was 9.22% and the saturated intensity was 0.09 MW cm^−2^ (Figure 3b), and Cs_4_PbBr_6_/CsPbBr_3_ exhibited a modulation depth of 16.83% with a corresponding saturation intensity of 0.52 MW cm^−2^ (Figure 3c). Notably, the modulation depth of Cs_4_PbBr_6_/CsPbBr_3_ composites surpassed that of Cs_4_PbBr_6_, which can be attributed to the type‐I heterojunction architecture inherent in Cs_4_PbBr_6_/CsPbBr_3_. In the Type‐I heterostructure, photogenerated electrons and holes are confined within the same material, which facilitates their radiative recombination and thereby improves carrier recombination efficiency.^[^ ^41^, ^42^, ^43^ ^]^

### 2.3 Ultrafast Mode‐Locking Operations

An EDFL ring cavity was meticulously assembled to facilitate the mode‐locking operation at 1.5 µm. The schematic configuration of the fiber laser is delineated in Figure S4 (Supporting Information). The EDFL cavity embodies a conventional ring resonator configuration comprising a 0.3 m‐long erbium‐doped fiber (EDF, Er110‐4/125) characterized by a group velocity dispersion (GVD) of 21.9 ps^2^ km^−1^ at 1550 nm, an 18.5 m‐long single‐mode fiber (SMF‐28e) characterized by a GVD of −22.3 ps^2^ km^−1^ Additionally, it incorporates a polarization‐independent isolator, a polarization controller (PC), a wavelength division multiplexer operating at 980/1550 nm, and an optical coupler (OC) featuring a 10% tap ratio at 1550 nm. Consequently, the total length of the fiber resonator spans 18.8 meters, resulting in a net negative dispersion of −0.405 ps^2^. The laser diode employed for pump emission operates at a peak power of 450 mW at 976 nm. Notably, without the presence of the SA, the generation of ultrashort pulses was unachievable, no matter how adjusting the pump power from 0 to 450 mW or polarization directions across the entire range.

First, the dispersed Cs_4_PbBr_6_/CsPbBr_3_ was deposited onto a tapered optical fiber characterized by a radius of 9 µm and an intrinsic loss of 25%. This assembly was subsequently integrated into the laser cavity, facilitating the generation of mode‐locked pulses within the C‐band through meticulous adjustments of the pump power and the polarization state. Mode‐locking was achieved by fine‐tuning the PC when the pump power was increased to 196 mW. **Figure** **4**a shows that when the pump power was varied from 196 to 361 mW, the output power was increased linearly from 436 to 931 µW, and the corresponding single pulse energy increased from 0.04 to 0.085 nJ. Figure 4b depicts the conventional soliton mode‐locked pulse sequence, with a consistent interval of 91.3 ns between neighboring pulses, aligning with the fundamental repetition frequency of 10.947 MHz. The soliton spectrum is shown in Figure 4c. The center wavelength is 1557 nm, and the 3 dB spectral width is 3.82 nm with clear Kelly sidebands. The asymmetry in the spectrum of traditional soliton mode‐locking may be attributed to Raman self‐frequency shift, interactions between solitons and intracavity dispersive waves, and the asymmetry of the gain spectrum.^[^ ^44^ ^]^ The autocorrelation trajectory is shown in Figure 4d. The pulse duration is 688 fs by using sech^2^ fitting. The calculated time‐bandwidth product (TBP) is 0.325, marginally exceeding the standard value for a transform‐limited pulse waveform (0.315), indicating a minor chirping effect present in the pulse. Figure 4e shows the radio‐frequency (RF) spectrum with a center frequency of 10.947 MHz and a signal‐to‐noise ratio (SNR) of ≈51.7 dB. The RF spectrum from 0 to 1 GHz is shown in Figure 4f, which exhibits good stability of the conventional soliton mode‐locked laser based on the Cs_4_PbBr_6_/CsPbBr_3_ SA.

The mode‐locking operation of pure Cs_4_PbBr_6_ was studied with the same experimental setup. The tapered fiber with Cs_4_PbBr_6_ SA has a radius of 8.6 µm and the same loss of 25%. In order to achieve stable mode‐locking of the laser pulse, the pump power was adjusted to 163.6 mW, resulting in the generation of a mode‐locked pulse from the EDFL at ≈1530 nm. The oscilloscope displayed a pulse sequence, as depicted in Figure S5b (Supporting Information), with an interval of 91.6 ns that corresponds to the cavity length of 18.8 m. The traditional soliton mode‐locked spectrum is presented in Figure S5c (Supporting Information), where symmetric Kelly sidebands are observed on both sides of the spectrum with a central wavelength of 1531.2 nm and a bandwidth of 4.35 nm.^[^ ^45^ ^]^ The continuous light observed at the upper end of the spectrum arises from the interplay between the fiber's birefringence effect and the nonlinear optical characteristics of the Cs_4_PbBr_6_ SA.^[^ ^46^, ^47^ ^]^ The autocorrelation trace measured by an autocorrelator indicated that the mode‐locked pulse has a width of 642 fs when fitted with a sech^2^ function (Figure S5d, Supporting Information). The TBP value of 0.357 suggested a small chirp observed within the laser cavity for the laser operation. Figure S5e,f (Supporting Information) shows the RF spectrum for a single RF and the 0–1 GHz range, revealing an SNR of ≈60 dB.

**Figure 5:**
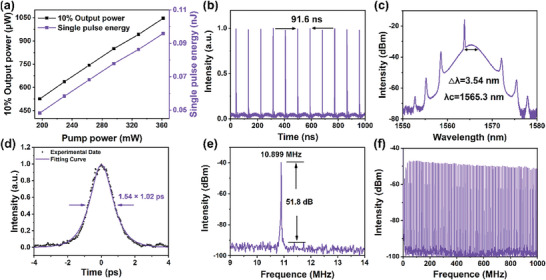
Mode‐locking of Cs_4_PbBr_6_ near 1560 nm. a) Variation of output power and single pulse energy; b) Pulse train; c) Typical optical spectrum; d) Autocorrelation trace; e,f) RF spectrum and RF spectrum with the range of 0–1 GHz.

As the pump power was gradually increased, we finely adjusted the PC to an optimal position, effectively managing the intracavity nonlinearity and birefringence. Upon reaching a pump power of 196.7 mW, this meticulous adjustment enabled us to achieve a conventional soliton mode‐locked operation near 1560 nm. In **Figure** **5**a, the relationship between output power and single pulse energy with pump power is illustrated. The highest recorded output power reached 1.064 mW at a pump power of 361.8 mW, corresponding to a single pulse energy of 0.096 nJ. Figure 5b displays the pulse sequence, depicting a pulse interval of 91.6 ns. Additionally, in Figure 5c, a characteristic spectrum of the mode‐locking is presented, revealing evident Kelly sidebands. The 3 dB spectral bandwidth is ≈3.54 nm and the peak central wavelength is 1565.3 nm in the steady‐state mode. As shown in Figure 5d, the observed autocorrelation trace has a pulse duration of 1.02 ps, which was obtained by a sech^2^ fit and the TBP is 0.42. RF spectrum analysis reveals a clear fundamental peak at 10.899 MHz corresponding to the ring laser cavity length (Figure 5e), and SNR for fundamental solitons exceeds 51.8 dB. A wide band RF range from 0 to 1 GHz displays equally spaced peaks validating fiber laser stability (Figure 5f).

By increasing the pump power to 229.9 mW and adjusting the PC, stable dual‐wavelength mode‐locking pulses were successfully attained. As depicted in Figure S6a (Supporting Information), as the pump power varies from 229.7 to 361.8 mW, the output power exhibits a linear increase from 595 to 1063 µW, alongside a corresponding rise in single pulse energy from 0.055 to 0.096 nJ. In Figure S6b (Supporting Information), the diagram illustrates the laser pulse sequence, revealing a pulse interval of 91.6 ns, which matches the length of the cavity. The spectrum chart is shown in Figure S6c (Supporting Information). There was a continuous wave pulse at 1531.9 nm. The central wavelength of the mode‐locked pulse was 1565.4 nm and the bandwidth was 2.8 nm. It had obvious Kelly sidebands and operated in the traditional soliton mode‐locking regime. After the sech^2^ fitting, a pulse duration of 985 fs was determined (Figure S6d, Supporting Information). Due to the dual‐wavelength effect, the single pulse underwent compression in comparison to the scenario with a single wavelength.^[^ ^48^ ^]^ The TBP was calculated by 0.337, which was a little larger than the transform‐limited value of hyperbolic secant. The RF exhibited a prominent signal peak, boasting a signal‐to‐noise ratio (SNR) of ≈57.8 dB. Concurrently, the mode‐locked fundamental repetition rate stands at 10.899 MHz, aligning with a cavity length of 18.8 m. As shown in Table S2 (Supporting Information), this study summarizes the performance of EDFLs based on Cs_4_PbBr_6_ and Cs_4_PbBr_6_/CsPbBr_3_ and compares their performance with different types of 2D materials and perovskite materials. The results indicate that EDFLs using Cs_4_PbBr_6_ and Cs_4_PbBr_6_/CsPbBr_3_ as SAs exhibit output performance that can be comparable to, or even surpassing, that of other semiconductor material lasers in terms of modulation depth, 3 dB bandwidth, and the pulse width. Moreover, Cs_4_PbBr_6_ and Cs_4_PbBr_6_/CsPbBr_3_ powders prepared directly via a one‐step method, offer additional advantages of low cost, simple preparation, and easy storage.

## 3 Conclusion

To summarise, pure Cs_4_PbBr_6_ and Cs_4_PbBr_6_/CsPbBr_3_ composites perovskites were synthesized utilizing a one‐step solution method. In the C‐band, pure Cs_4_PbBr_6_ possessed a modulation depth of 9.22% and a saturation intensity of 0.09 MW cm^−2^. The modulation depth of Cs_4_PbBr_6_/CsPbBr_3_ was 16.83% and the saturated intensity was 0.52 MW cm^−2^, which was larger than that of Cs_4_PbBr_6_. This enhancement can be attributed to the increased carrier mobility inherent to heterostructures. This underscores the favorable nonlinear optical characteristics inherent in Cs_4_PbBr_6_ and Cs_4_PbBr_6_/CsPbBr_3_‐based saturable absorbers. Stable mode‐locking operations were observed with the incorporation of both saturable absorbers into an EDFL system. These findings highlighted the potential of 0D Cs_4_PbBr_6_ and Cs_4_PbBr_6_/CsPbBr_3_ composites as promising candidates for facilitating the generation of mode‐locked laser pulses within the C‐band spectral range. Furthermore, the effectiveness in perovskites suggests significant potential for exploitation in the broadband nonlinear optical response regime.

## 4 Experimental Section

### Chemical for Preparing Cs_4_PbBr_6_/CsPbBr_3_ and Cs_4_PbBr_6_

PbBr_2_ (99.9%, Macklin), CsBr (99.9%, Aladdin), Dimethyl Sulfoxide (DMSO, >99.8%(GC), Macklin), toluene (≥99.5%, Sinopharm Chemical Reagent Co., Ltd.). All chemicals were used without further purification.

### Preparation of Perovskites by a One‐Step Solution Method

1 mmol of PbBr_2_ and 1 mmol of CsBr were dissolved in a beaker containing 3 mL of DMSO. The mixed solution was heated on a heating table at 100 °C and stirred thoroughly until completely dissolved. Then 5 mL of toluene was added to the heated solution as an anti‐solvent to force precipitation, and the solution was stirred vigorously for at least 2 h to ensure complete solvent reaction. To extract the reaction product, the solution was kept stationary and the particles would rapidly sink to the bottom. The supernatant was discarded, the precipitate was washed with toluene and centrifuged, and the centrifugal washing process was repeated several times. Finally, the precipitate was dried using a vacuum drying oven to obtain Cs_4_PbBr_6_/CsPbBr_3_ powder. The preparation of pure Cs_4_PbBr_6_ was similar to that of Cs_4_PbBr_6_/CsPbBr_3_ powder, but DMSO was increased to 5 mL, while the other steps were consistent.

### Characterization

Scanning electron microscopy (Quanta FEG 250) is employed for observing the morphology of the sample. The phase of the material was studied using an X‐ray diffractometer (Rigaku Miniflex 600, Japan). The UV–vis–IR spectrum was tested by Shimadzu UV‐3600, Japan. XPS measurements were performed on Thermo Scientific K‐Alpha, America. A mode‐locked pulse train was recorded with an oscilloscope (MDO4104C, Tektronix Inc.), a spectrum analyzer (6375D, Yokogawa Inc.) to test the spectrum and an autocorrelator were used to analyze the pulse width (FR‐103XL, Femtochrome Inc.) and a spectrum signal analyzer to test the Radio frequency spectrum (FPC1000, Rohde & Schwarz Inc.).

## Conflict of Interest

The authors declare no conflict of interest.

## Author Contributions

C.H. and J.N. designed and performed the experiments. P.H. and P.Z. performed the formal analysis. Z.S. helped analyze the data. C.H. and L.D. conceived the idea and co‐supervised the project. All authors contributed to the general discussion.

## Supporting information

Supporting Information

## Data Availability

The data that support the findings of this study are available from the corresponding author upon reasonable request.
