# Supplementary material for: One‐Step Fabrication of 0D Cs4PbBr6 Perovskite with Nonlinear Optical Properties for Ultrafast Pulse Generation
Source: Adv Sci (Weinh). 2024 Jul 12;11(35):2404465. doi: 10.1002/advs.202404465 (PMC11425289; doi:10.1002/advs.202404465)
Supplement: Supplementary file 1 — Supporting Information [file ADVS-11-2404465-s001.docx]

**Supporting Information**

**One-step fabrication of zero-dimensional Cs_4_PbBr_6_ perovskite with nonlinear optical properties for ultrafast pulse generation**

Ning Jiang, Hongwei Chu*, Zhongben Pan, Han Pan, Shengzhi Zhao and Dechun Li*

N. Jiang, H. Chu, Z. Pan, H. Pan, S. Zhao, D. Li

School of Information Science and Engineering

and Key Laboratory of Laser and Infrared System of Ministry of Education

Shandong University

Qingdao 266237, China

E-mail: hongwei.chu@sdu.edu.cn; dechun@sdu.edu.cn


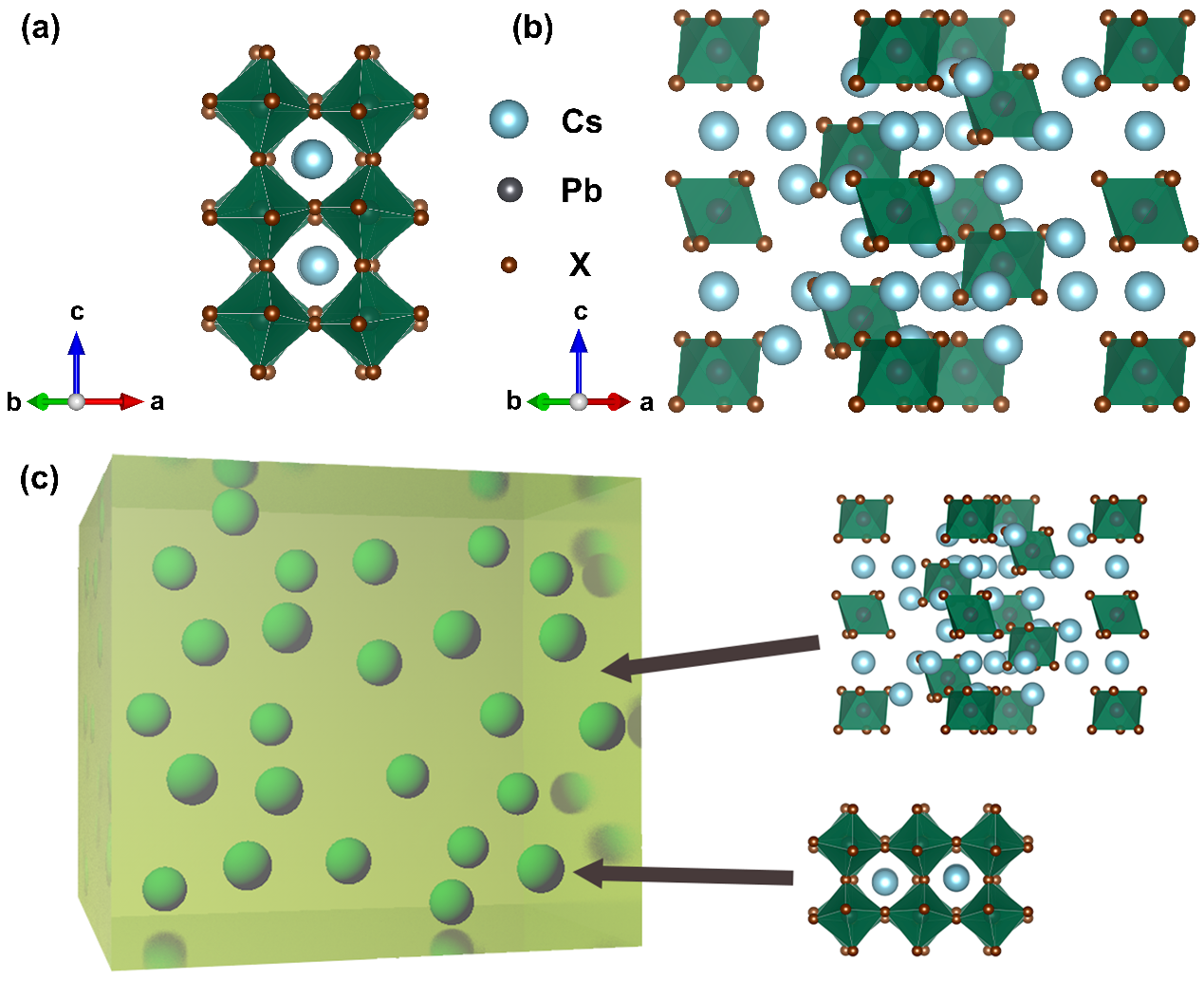


**Figure S1.** Crystal structure of (a) CsPbX_3_ and (b) Cs_4_PbX_6_; (c) The crystal structure diagram of Cs_4_PbBr_6_/CsPbBr_3_ composites.


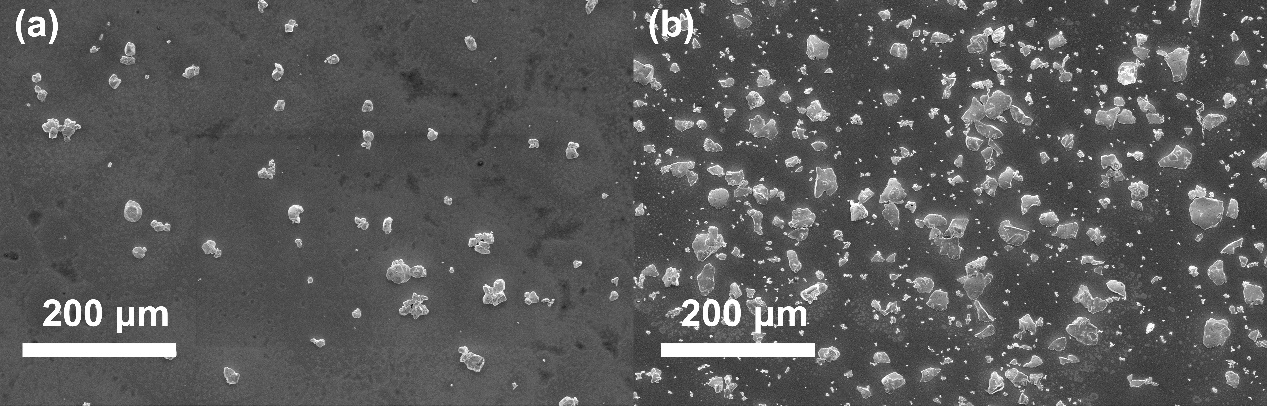


**Figure S2.** SEM images of mixed-phase Cs_4_PbBr_6_/CsPbBr_3_ (a) and Cs_4_PbBr_6_ (b) powders after dispersion.

Table S1 the ICP-MS of Cs_4_PbBr_6_ and Cs_4_PbBr_6_/CsPbBr_3_ composites.

|  | | Cs_4_PbBr_6_ | Cs_4_PbBr_6_/CsPbBr_3_ |
| --- | --- | --- | --- |
| Atomic (%) | Cs | 36.14 | 34.67 |
|  | Pb | 9.24 | 10.25 |
|  | Br | 54.62 | 55.08 |
| Cs: Pb | | 3.91 | 3.38 |
| Cs_4_PbBr_6_: CsPbBr_3_ | | 0.97:0.03 | 0.79:0.21 |


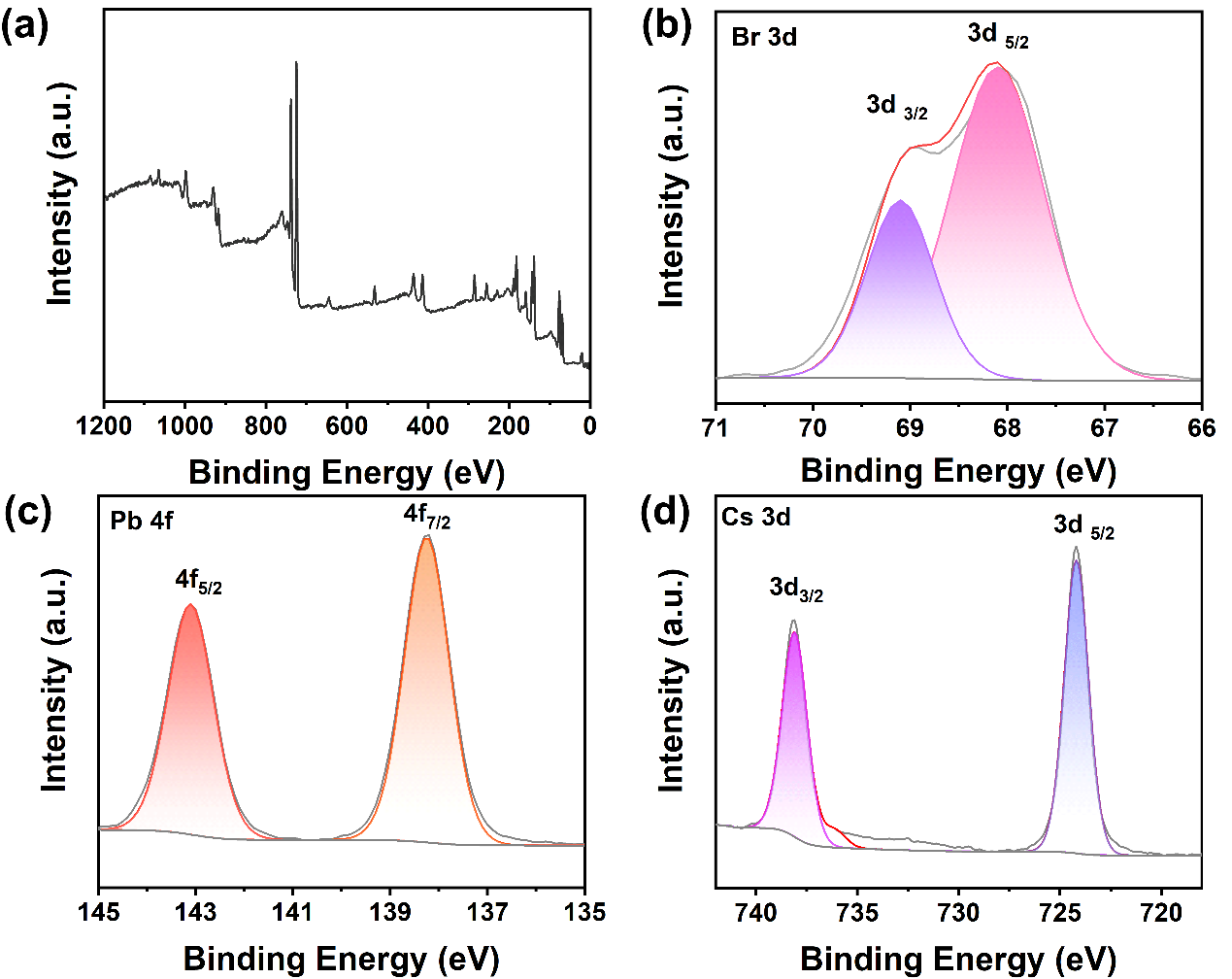


**Figure S3.** XPS spectrum of Cs_4_PbBr_6_/CsPbBr_3_ powders.


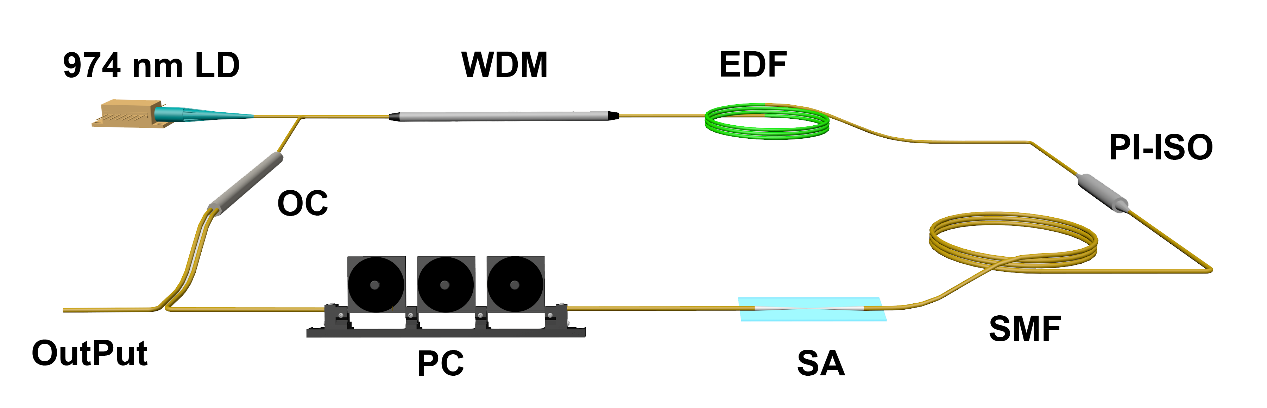


**Figure S4.** Structural diagram for the conventional soliton mode-locking operations.


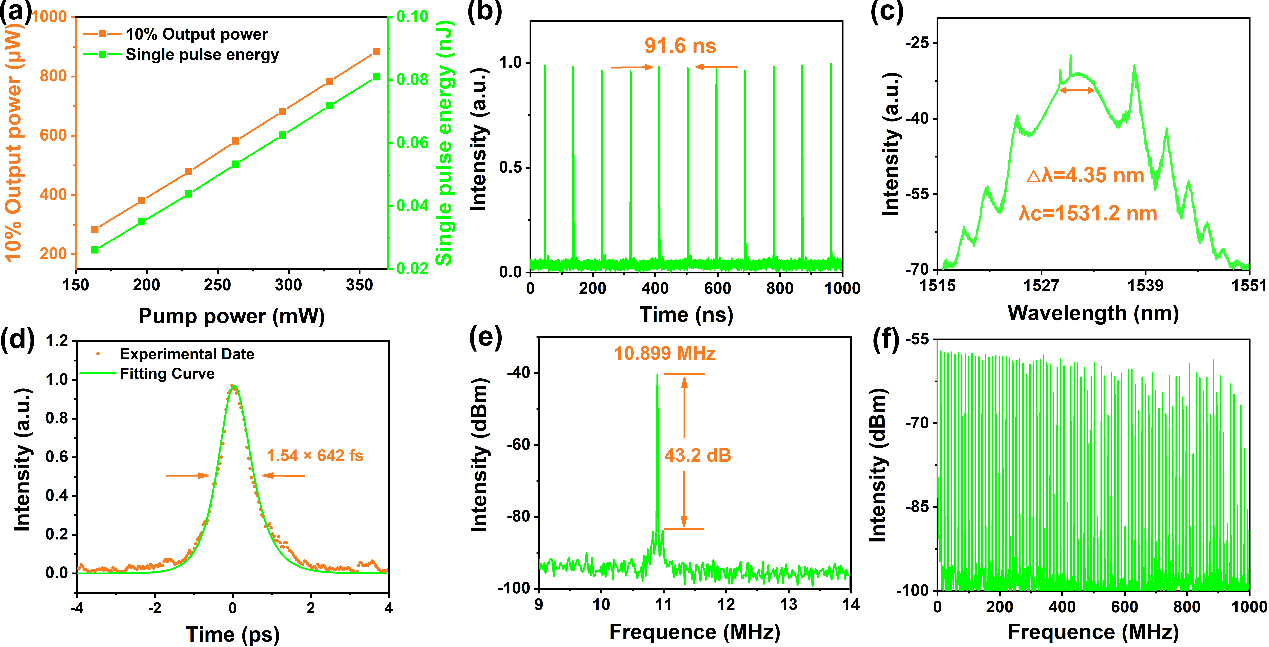


**Figure S5.** Mode-locking of Cs_4_PbBr_6_ near 1530 nm (a) Variation of output power and single pulse energy; (b) Pulse train; (c) Typical optical spectrum; (d) Autocorrelation trace; (e-f) Radiofrequency spectrum and radio frequency spectrum with the range of 0-1GHz.


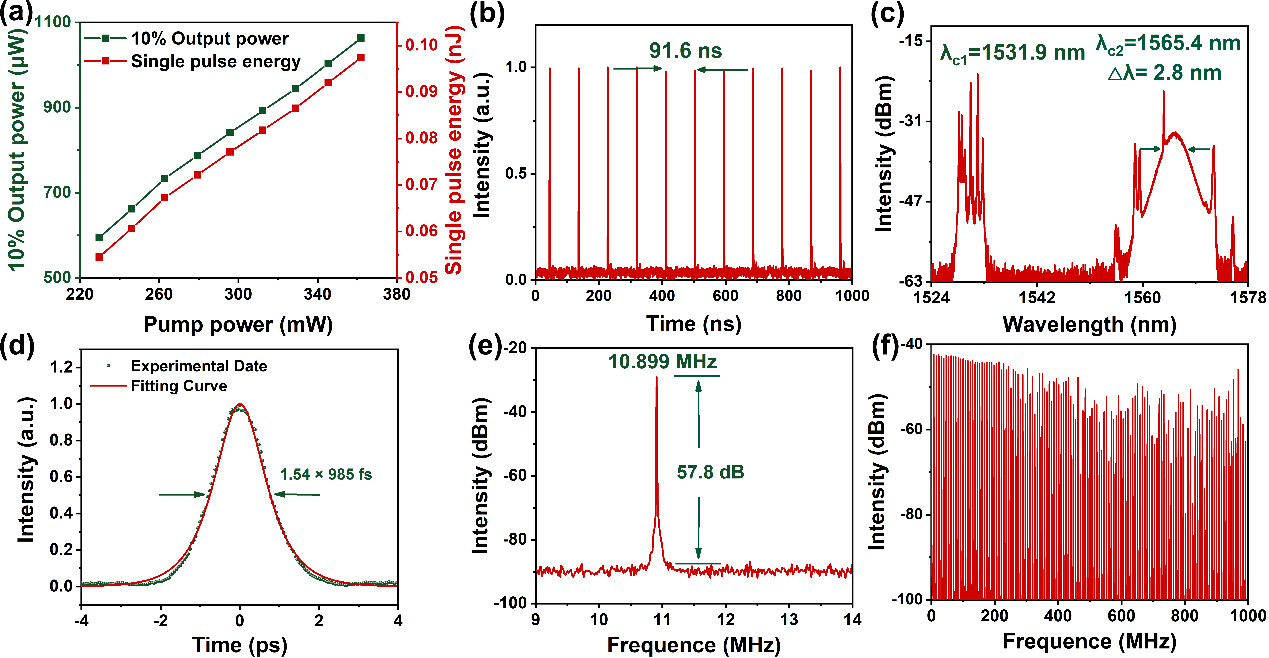


**Figure S6.** Synchronized mixed-wavelength mode-locking of Cs_4_PbBr_6_ near 1530 and 1560nm (a) Variation of output power and single pulse energy; (b) Pulse train; (c) Typical optical spectrum; (d) Autocorrelation trace; (e) RF spectrum; (f) Wide-band RF spectrum.

Table S2. Performance Summary of Mode-Locked Fiber Lasers Operating at 1.5 μm by Using Different Types of Materials as SAs

| Material | Modulation Depth (%) | Center Wavelength (nm) | 3dB Δλ (nm) | Pulsed Width  (ps) | Ref. |
| --- | --- | --- | --- | --- | --- |
| Black phosphorus | 8.1 | 1571.45 | 2.925 | 0.946 | [1] |
| Bismuth | 3 | 1569.04 | 4.2 | 0.645 | [2] |
| Graphite | 20 | 1568 | 1.58 | 1.67 | [3] |
| CuO | 5.8 | 1565.8 | 2.2 | 1.26 | [4] |
| PbS | 12.2 | 1533 | 2.93 | 1.01 | [5] |
| WS_2_ | 15.1 | 1568.3 | 1.94 | 1.49 | [6] |
| CsCu_2_I_3_ | 12.04 | 1561 | 5.83 | 0.515 | [7] |
| CH_3_NH_3_PbI_3_ | 9.6 | 1568.9 | 2.75 | 1.75 | [8] |
| Cs_4_PbBr_6_/CsPbBr_3_ | 16.83 | 1557 | 3.82 | 0.688 | This Work |
| Cs_4_PbBr_6_ | 9.22 | 1531.2 | 4.35 | 0.642 |  |
|  |  | 1565.3 | 3.54 | 1.02 |  |
|  |  | 1531.9 (CW), 1565.4 | 2.8 | 985 |  |

**Reference**

[1] Y. Chen, G. Jiang, S. Chen, Z. Guo, X. Yu, C. Zhao, H. Zhang, Q. Bao, S. Wen, D. Tang, D. Fan, *Opt. Express* **2015**, 23, 12823.

[2] L. Du, D. Lu, J. Li, K. Yang, L. Yang, B. Huang, J. Yi, Q. Yi, L. Miao, X. Qi, C. Zhao, J. Zhong, S. Wen, *ACS Appl. Mater. Interfaces* **2019**, 11, 35863.

[3] Y. H. Lin, G. R. Lin, *Laser Phys. Lett.* **2012**, 9, 398.

[4] Y. Zhao, W. Wang, X. Li, H. Lu, Z. Shi, Y. Wang, C. Zhang, J. Hu, G. Shan, *ACS Photonics* **2020**, 7, 2440.

[5] Y. Zhang, X. Li, A. Qyyum, T. Feng, P. Guo, J. Jiang, H. Zheng, *Part. Part. Syst. Char.* **2018**, 35, 1800341.

[6] P. Yan, H. Chen, J. Yin, Z. Xu, J. Li, Z. Jiang, W. Zhang, J. Wang, I. L. Li, Z. Sun, S. Ruan, *Nanoscale* **2017**, 9, 1871.

[7] H. Deng, X. Xu, F. Liu, Q. Yu, B. Shu, Z. Yang, S. Zhu, Q. Zhang, J. Wu, P. Zhou, *J. Mater. Chem. C* **2023**, 11, 1696.

[8] L. Miao, G. Jiang, L. Du, B. Huang, W. Hu, C. Zhao, S. Wen, *IEEE Photonics Technol. Lett.* **2018**, 30, 577.
